# Enhanced Electrochemical Performances of Cobalt-Doped Li_2_MoO_3_ Cathode Materials

**DOI:** 10.3390/ma12060843

**Published:** 2019-03-13

**Authors:** Zhiyong Yu, Jishen Hao, Wenji Li, Hanxing Liu

**Affiliations:** 1 State Key Laboratory of Advanced Technology for Materials Synthesis and Processing, Wuhan University of Technology, Wuhan 430070, China; haojishen@live.com (J.H.); lwjwhut@126.com (W.L.); 2 School of Materials Science and Engineering, Wuhan University of Technology, Wuhan 430070, China; 3 International School of Materials Science and Engineering, Wuhan University of Technology, Wuhan 430070, China

**Keywords:** Li_2_MoO_3_, Co-doping, cathode materials, Li ion battery

## Abstract

Co-doped Li_2_MoO_3_ was successfully synthesized via a solid phase method. The impacts of Co-doping on Li_2_MoO_3_ have been analyzed by X-ray photoelectron spectroscopy (XPS), X-ray powder diffraction (XRD), scanning electron microscope (SEM), and Fourier transform infrared spectroscopy (FTIR) measurements. The results show that an appropriate amount of Co ions can be introduced into the Li_2_MoO_3_ lattices, and they can reduce the particle sizes of the cathode materials. Electrochemical tests reveal that Co-doping can significantly improve the electrochemical performances of the Li_2_MoO_3_ materials. Li_2_Mo_0.90_Co_0.10_O_3_ presents a first-discharge capacity of 220 mAh·g^−1^, with a capacity retention of 63.6% after 50 cycles at 5 mA·g^−1^, which is much better than the pristine samples (181 mAh·g^−1^, 47.5%). The enhanced electrochemical performances could be due to the enhancement of the structural stability, and the reduction in impedance, due to the Co-doping.

## 1. Introduction

Recently, the development of high-capacity cathode materials has become a hot topic in the field of Li-ion batteries. Mn-based Li-rich layer oxides *x*Li_2_MnO_3_·(1 − *x*)LiMO_2_ (0 < *x* < 1.0, M = Mn, Ni, Co, etc.) were proposed as potential cathode materials, due to their high discharge capacities of above 280 mAh·g^−1^, and thus, the structure stability of the Li_2_MnO_3_ component [1,2,3,4,5,6,7,8]. Unfortunately, numerous reports have indicated that the drawbacks of Li_2_MnO_3_-based composites, such as low initial Coulombic efficiency, a fast decline in capacity, and potential safety hazards, were difficult to overcome, which severely restricted their practice applications [9,10,11]. Thus, much attention has been paid to find other transition metals instead of Mn, to build new Li_2_MO_3_ (M = Ru, Ir, Mo, etc.)-based materials for next generation Li–ion batteries in recent years [12,13,14,15].

Li_2_MoO_3_ as a type of Li–rich layer cathode material with alternating Li layers and randomly distributed [Li_1/3_Mo_2/3_] layers, has attracted much research interest [15,16,17,18,19,20,21]. The previous studies verified that Li_2_MoO_3_ promised a high theoretical capacity of up to 339 mAh·g^−1^, and a near-absence of oxygen evolution [17,18], which supported Li_2_MoO_3_ as a candidate to replace Li_2_MnO_3_ in constructing Li-rich cathode materials. However, the poor cycling stability and rate capability of the Li_2_MoO_3_ material, owing to its low conductivity and irreversible phase transition, hinders its practical application. Hence, it is necessary to find a suitable modification method to improve the performance of the Li_2_MoO_3_ material. 

At present, only a few studies about on modifying Li_2_MoO_3_ have been reported [19,20,21]. Ceder’s group constructed a solid solution between Li_2_MoO_3_ and LiCrO_2_ for cathode materials [19]. The Li_2_MoO_3_–LiCrO_2_ cathode materials presented not only high-discharge capacities, but also great cycling stabilities over the 10 cycles. In our previous study, carbon-coated Li_2_MoO_3_ composites were successfully prepared, and they achieved much lower impedances and better electrochemical performances than bare Li_2_MoO_3_ [21]. Cobalt doping has been considered to be a facile and effective method in enhancing the electrochemical performances, since it can improve structure stability and reduce the impedance of cathode materials [22,23,24,25,26]. In this paper, cobalt was selected to improve the electrochemical performances of Li_2_MoO_3_ for the first time. The structural characteristics and electrochemical performances of Li_2_Mo_1−*x*_Co*_x_*O_3_ are presented here. 

## 2. Materials and Methods 

### 2.1. Preparation of the Li_2_Mo_1−x_Co_x_O_3_ Powder

**Figure 1:**
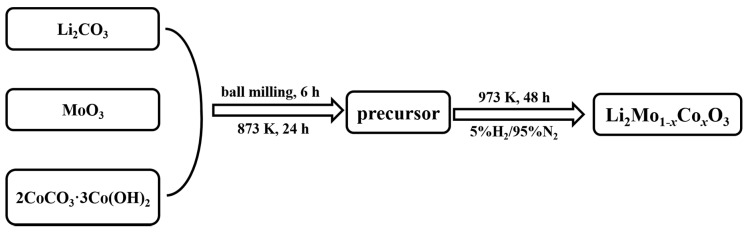
Flow chart of the process for preparing Li_2_Mo_1−*x*_Co*_x_*O_3_.

The pristine and Co-doped Li_2_MoO_3_ powders were synthesized via a solid reaction method, as shown in Figure 1. Firstly, stoichiometric amounts of Li_2_CO_3_ (>99.7%, Sinopahrm Medicine, Shanghai, China), MoO_3_ (>99.5%, Aldrich, Shanghai, China), and 2CoCO_3_·3Co(OH)_2_ (>99.5%, Aldrich, Shanghai, China) were homogeneously mixed by ball milling, and then calcinated at 873 K for 24 h under air, to obtain the precursor. Li_2_CO_3_ was added in at 10% excess to compensate Li volatilization. After that, the obtained precursor was reduced in a stream of flowing 5%H_2_/95%N_2_ at 973 K for 48 h to prepare Li_2_Mo_1−*x*_Co*_x_*O_3_.

### 2.2. Physical Characterization

X-ray powder diffraction (XRD) was carried out by using a PhilipsX’ Pert PW3050/60 diffractometer (PANalytical. B. V, Lelyweg, the Netherlands), with a scan rate of 0.02° per second, by Cu–Kα radiation (λ = 1.5406 Å). X’pert Highscor software (PANalytical. B. V, Lelyweg, the Netherlands) was used for Rietveld refinement. The morphologies of the samples was determined by a HITACHI S-4800 field-emission high-resolution scanning electron microscope (SEM) (Hitachi, Tokyo, Japan). X-ray photoelectron spectroscopy (XPS) was detected on a ESCALAB250Xi (ThermoFisher, Waltham, America). Fourier transform infrared spectroscopy (FTIR) was detected on Nicolet6700 (ThermoFisher, Waltham, America) in the wave range of 4000–400 cm^−1^ with a high resolution of 4 cm^−1^.

### 2.3. Electrochemical Tests

The electrochemical performances were tested through CR2032-type coin cells (HF-Kejing, Hefei, China). Cathodes were prepared by mixing 70 wt % Li_2_Mo_1−*x*_Co*_x_*O_3_, 20 wt % acetylene carbon black and 10 wt % polyvinylidene fluoride (PVDF) in *N*-methyl-2-pyrrolidone (NMP) solution. The slurry was cast evenly onto a stainless steel sheet, and dried in a vacuum oven (Suopuyiqi, Shanghai, China) at 120 °C for 12 h. Lithium metal and Celgard 2400 were used as the anode and separator, respectively. A concentration of 1 mol·L^−1^ LiPF_6_ in ethylene carbonate (EC)/dimethyl carbonate (DMC) (volume ratio: 1:1) solution was adopted as the electrolyte. The coin cells were assembled in an argon-filled glove box, and measured on a Land CT2001A system (LANHE, Wuhan, China) in galvanostatic mode at 30 °C. Electrochemical impedance spectroscopy (EIS) was tested using an electrochemical station (CHI660B) (Chenhua, Shanghai, China) in 10^−2^ Hz–10 MHz, with a voltage amplitude of 5 mV. Cycliation at a scanning speed of 10^−1^ mVc voltammetry (CV) was performed with the same electrochemical st s^−1^.

## 3. Results and Discussion

### 3.1. Characteristics of the as-Prepared Li_2_Mo_1−x_Co_x_O_3_

**Figure 2:**
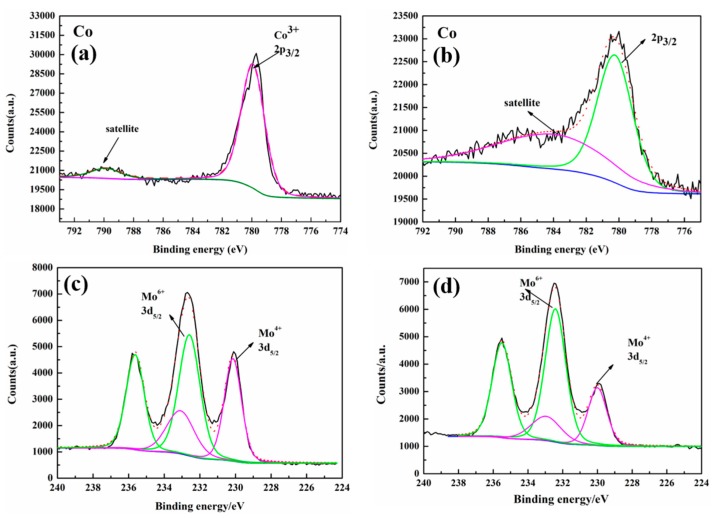
XPS spectra of Co in (**a**) the doped precursor, and (**b**) Li_2_Mo_0.90_Co_0.10_O_3_ and the XPS spectra of Mo in (**c**) Li_2_MoO_3_ and (**d**) Li_2_Mo_0.90_Co_0.10_O_3_.

Figure 2a and b shows the XPS spectra of Co 2p_3/2_ in Li_2_Mo_0.90_Co_0.10_O_3_, and the corresponding precursor. The peak of Co 2p_3/2_ in the precursor is at 779.8 eV (Figure 2a) with a weak satellite at 789.9 eV. This satellite peak is 10.1 eV above Co 2p_3/2_, corresponding diamagnetic Co^3+^ (S = 0), which is very consistent with the XPS result reported for LiCoO_2_ [27]. After the reduction by hydrogen at a high temperature, the original satellite disappears, and a new intense satellite appears at 784.4 eV, as shown in Figure 2b. Compared with the original satellite, the new satellite is 4.1 eV higher than the core level line (780.3 eV), corresponding to high-spin Co^2+^ (S = 3/2) compounds, indicating that the valence state of the Co element will be reduced from Co^3+^ to Co^2+^ after reduction processing under hydrogen [28]. Figure 2c and d show that the XPS spectra of Mo in Li_2_MoO_3_ and Li_2_Mo_0.90_Co_0.10_O_3_. The peak at around 230.1 eV is assigned to Mo^4+^ 3d_5/2_, and the peak at around 232.6 eV is assigned to Mo^6+^ 3d_5/2_. Obviously, after cobalt doping, the peak intensity of Mo^6+^ 3d_5/2_ rises, and the peak intensity of Mo^4+^ 3d_5/2_ decreases, indicating an increased amount of Mo^6+^.

**Figure 3:**
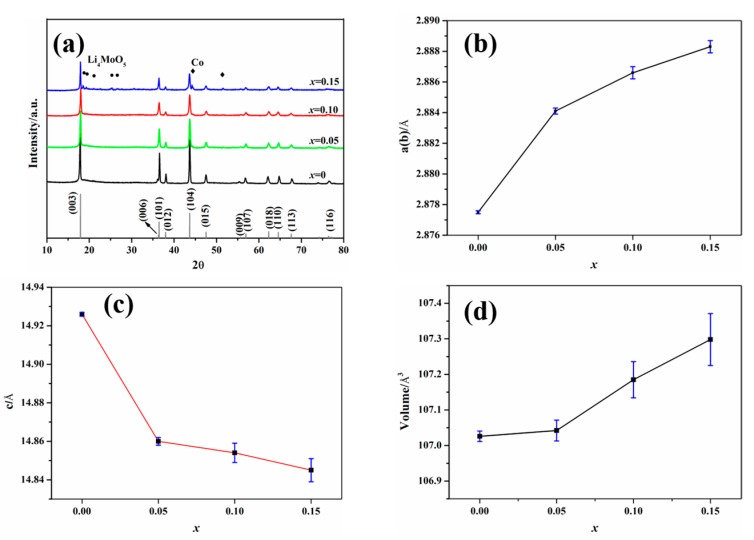
(**a**) XRD patterns, (**b**) a(b)-parameters in the lattice, (**c**) c-parameters, and (**d**) unit cell volume of the synthesized Li_2_Mo_1−*x*_Co*_x_*O_3_ (*x* = 0, 0.05, 0.10, 0.15).

Figure 3a shows the XRD patterns of the synthesized Li_2_Mo_1−*x*_Co*_x_*O_3_ (*x* = 0, 0.05, 0.10, 0.15). Except the sample with *x* = 0.15, all samples match well with the α-NaFeO_2_ structure, which could be indexed to Li_2_MoO_3_ (see Figure 3a). When the Co content adds up to 0.15, the characteristic peaks of the impurity phase Li_4_MoO_5_ and Co appear. The splitting of the (006)/(101) peaks at 36°, reflecting that the layer structure weakens with the increase of Co content, indicating that Co-doping increases the disorder of the cations. Rietveld refinements for Li_2_Mo_1−*x*_Co*_x_*O_3_ were carried out, to obtain more information from XRD (see Figure 3b,c). The a(b)-parameters are increased, and c-parameters are decreased, with a rise of Co-doping content. Notably, the variations of cell parameters are negligible while *x* is higher than 0.10 considering the fitting error, which indicates that the solubility limit of Co is around *x* = 0.10. Moreover, the values of c/a drop with the increase of the Co-doping content, which indicates that Co-doping increases the disorder of Li_2_MoO_3_ materials. As indicated in the above XPS results, the valence of cobalt should be +2 in the samples. Its radius (0.745 Å) is very similar to that of Li^+^ (0.76 Å), which may result in the increase of disorder. Figure 3d exhibits the unit cell volume of the pristine and the Co-doped Li_2_MoO_3_. Clearly, the unit cell volume increases with the rise of the Co-doping contents, which could be related to the replacement of Mo^4+^ (0.65 Å) by Co^2+^ (0.745 Å). 

**Figure 4:**
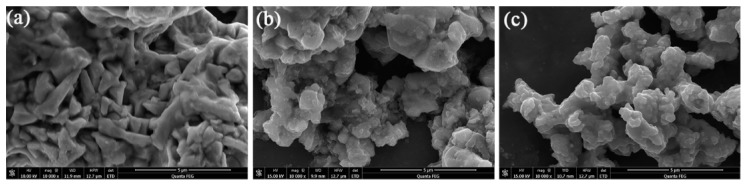
The SEM images of (**a**) Li_2_MoO_3_, (**b**) Li_2_Mo_0.95_Co_0.05_O_3_ and (**c**) Li_2_Mo_0.90_Co_0.10_O_3_.

**Figure 5:**
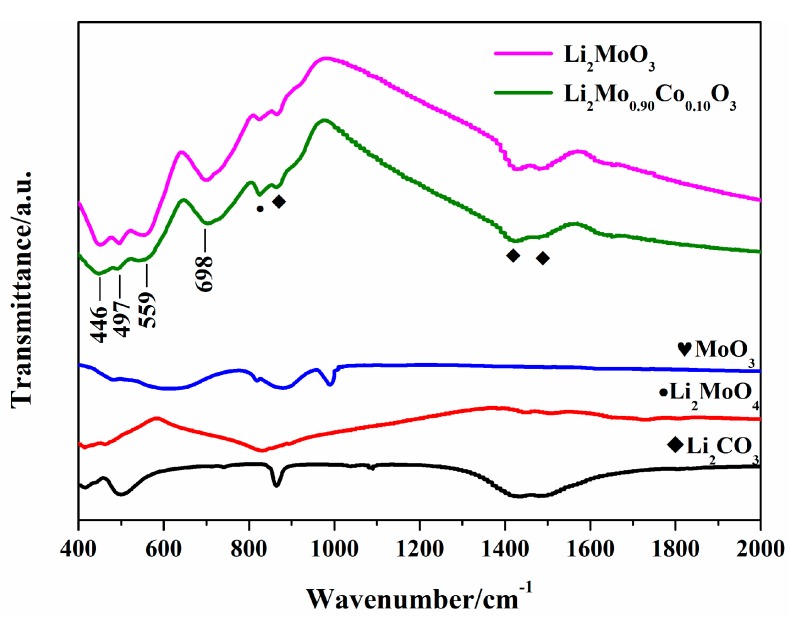
The FTIR spectra of Li_2_MoO_3_ and Li_2_Mo_0.90_Co_0.10_O_3_.

In order to observe the impacts of Co-doping on the particle morphologies of the samples, the SEM images of Li_2_Mo_1−*x*_Co*_x_*O_3_ (*x* = 0, 0.05, 0.10) were examined (see Figure 4). The particle sizes of the pristine Li_2_MoO_3_ present a wide distribution range from 1 to 3 µm (see Figure 4a), whereas the doped samples show smaller particles with more uniform distributions in the range of 200–300 nm (see Figure 4b,c). The results suggest that the addition of Co affects the morphology, and decreases the particle size of cathode materials. Particle growth may be restricted by lattice distortion of Li_2_MoO_3_ due to the replacement of Mo by Co. Similar phenomena have also been observed by some other groups [29,30]. Ma, J. et al. studied the stability of Li_2_MoO_3_ in air [16]. Their results verified that Li_2_MoO_3_ easily adsorbed O_2_ and thus was partially oxidized to Li_2_MoO_4_. Meanwhile, the CO_2_ in air also reacted with Li_2_MoO_3_ to produce Li_2_CO_3_, which consumed the Li ions near the surface and produced MoO_3_ [16]. In order to investigate the effects of Co-doping on the stability of Li_2_Mo_1−*x*_Co*_x_*O_3_ in air, FTIR spectra of Li_2_MoO_3_ and Li_2_Mo_0.90_Co_0.10_O_3_ were carried out (see Figure 5). The samples were stored in the air for 7 days before the FTIR test. Obviously, both samples present a similar FTIR spectra. The peaks at 446, 497, 559 and 698 cm^−1^ are assigned to Li_2_MoO_3_, which is consistent with the previous study [16]. While the peaks at 1480, 1420, 830 and 817 cm^−1^ could be attributed to Li_2_CO_3_ and Li_2_MoO_4_, respectively. These species are believed to be the reaction products between of Li_2_MoO_3_, CO_2_ and O_2_, revealing that both samples are partially decomposed in air. No peaks related MoO_3_ are detected in the FTIR spectra, which may contribute to the shorter storage time compared with the previous report [16].

### 3.2. Electrochemical Performances

**Figure 6:**
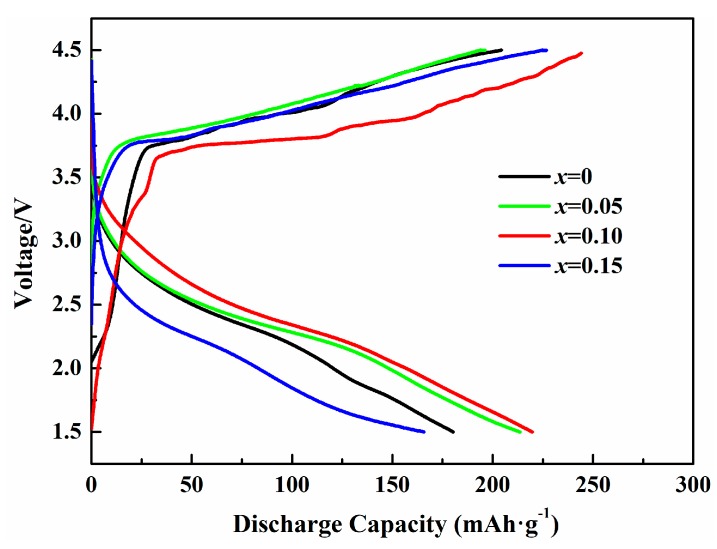
The initial charge–discharge profiles of the synthesized Li_2_Mo_1−*x*_Co*_x_*O_3_ (*x* = 0, 0.05, 0.10, 0.15) between 1.5 and 4.5 V at 5 mA·g^−1^.

Figure 6 compares the initial charge-discharge profiles of pristine and Co-doped samples in the voltage range of 1.5–4.5 V at 5 mA·g^−1^. The samples present initial discharge capacities of 181, 213, 220 and 165 mAh·g^−1^, respectively. When the doping content is 0.05 and 0.10, the first discharge capacities of Co-doped samples are higher than that of the pristine sample. It also can be found the first discharge capacity decreases obviously while *x* = 0.15. What’s more, voltage difference between charge and discharge profiles of Li_2_Mo_0.90_Co_0.10_O_3_ is much smaller than that of pristine Li_2_MoO_3_, indicating that Co-doping can effectively suppress the polarization and enhance reversibility of Li_2_MoO_3_ materials. Notable that the charge behaviors with two regions for Co-doped Li_2_MoO_3_ cathode materials are similar to that for the pristine sample in the first charge-discharge process, which may relate to the delithiation reaction corresponding to the oxidation of the Mo ions in the 1.5–3.7 V region and a Li_2_MoO_3_-Li_0.91_MoO_3_ two phase reaction in the 3.7–4.5 V region [17]. 

**Figure 7:**
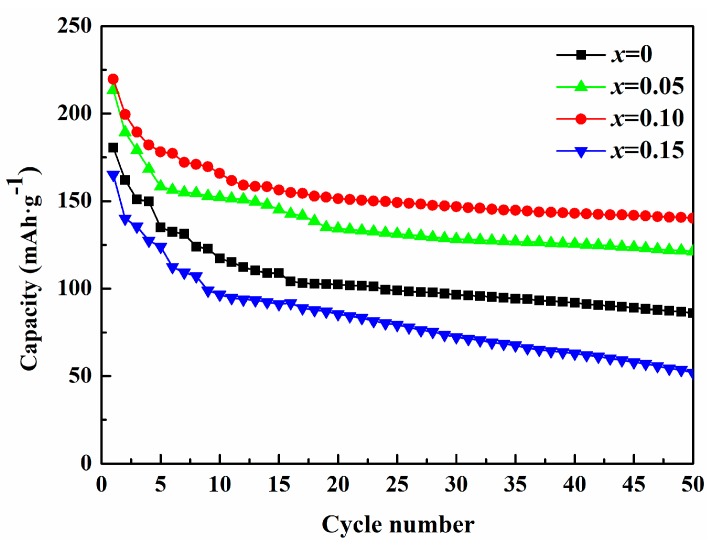
Cycling performances of the synthesized Li_2_Mo_1−*x*_Co*_x_*O_3_ (*x* = 0, 0.05, 0.10, 0.15) between 1.5 and 4.5 V at 5 mA·g^−1.^

Figure 7 shows the cycling performances of pristine and Co-doped samples between 1.5 and 4.5 V at 5 mA·g^−1^. It is clearly seen that Li_2_Mo_0.95_Co_0.05_O_3_ and Li_2_Mo_0.90_Co_0.10_O_3_ deliver much higher discharge capacity than that of pristine sample. After 50 cycles, pristine and Co-doped sample present the discharge capacities of 86, 121, 140 and 52 mAh·g^−1^ with the capacity retentions of 47.5%, 56.8%, 63.6% and 31.5%, respectively. Clearly, the cycling stability of pristine is poor. However, while increasing cobalt content to 0.05 and 0.10, the discharge capacity and cycling stability are significantly improved. With a further increase of Co content, the discharge capacity and cycling stability reduce, which may be attributed to the rise of inert impurities of Li_4_MoO_5_ and Co. Li_4_MoO_5_ delivers poor electrochemical performances because of its low electron conduction, low Coulombic efficiency and critical irreversible phase transition [31]. In addition, Co shows ignorable specific capacity above 1.5 V [32]. Therefore, the appearance of Co and Li_4_MoO_5_ in the sample has negative effects on the electrochemical performance of Li_2_MoO_3_. Li_2_Mo_0.90_Co_0.10_O_3_ possesses the highest discharge capacity and the best capacity retention, which indicates the amounts of dopant will be important for the electrochemical performances of Li_2_MoO_3_.

**Figure 8:**
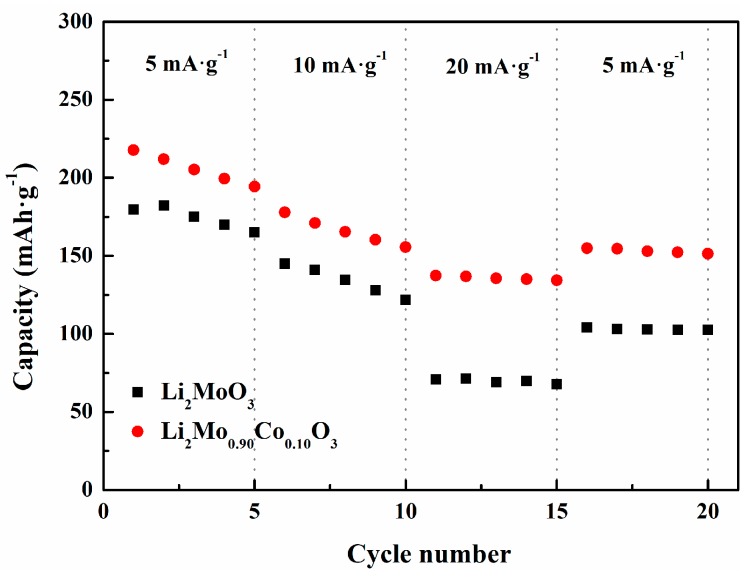
Rate performances of Li_2_MoO_3_ and Li_2_Mo_0.90_Co_0.10_O_3_ at different current density.

The comparison of rate capabilities between the pristine and Co-doped Li_2_MoO_3_ with current density from 5 mA·g^−1^ to 20 mA·g^−1^ is evaluated in Figure 8. The Li_2_Mo_0.90_Co_0.10_O_3_ possesses a higher discharge capacity of 218 mAh·g^−1^ at 5 mA·g^−1^ and 137 mAh·g^−1^ at 20 mA·g^−1^ than pristine Li_2_MoO_3_ material (180 mAh·g^−1^ at 5 mA·g^−1^ and 71 mAh·g^−1^ at 20 mA·g^−1^). The difference of discharge capacities between Li_2_MoO_3_ and Li_2_Mo_0.90_Co_0.10_O_3_ increases from 38 mAh·g^−1^ to 66 mAh·g^−1^ with a rise of current density from 5 mA·g^−1^ to 20 mA·g^−1^. When the current density returns to 5 mA·g^−1^, the discharge capacity of Li_2_Mo_0.90_Co_0.10_O_3_ could reach up 155 mAh·g^−1^, while that of pristine Li_2_MoO_3_ only lefts 104 mAh·g^−1^. The above results suggest that Co-doping significantly enhances the rate capability of Li_2_MoO_3_.

**Figure 9:**
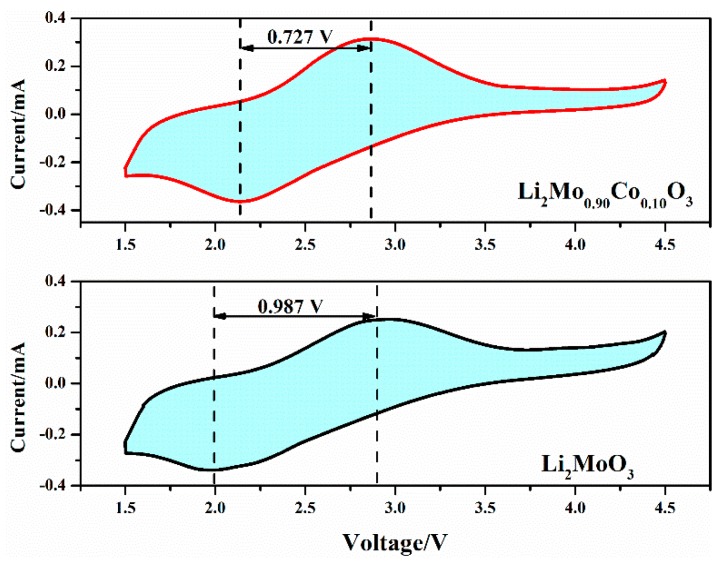
CV curves of Li_2_MoO_3_ and Li_2_Mo_0.90_Co_0.10_O_3_ between 1.5 and 4.5 V at the 5th cycle.

Figure 9 illustrates CV curves of Li_2_MoO_3_ and Li_2_Mo_0.90_Co_0.10_O_3_ between 1.5 and 4.5 V. The redox peaks of Li_2_MoO_3_ in CV curve are located at 2.965 V and 1.978 V, which are respectively related to the delithiation/lithiation processes corresponding to the oxidation/reduction of Mo^4+^/Mo^6+^ couple [18]. The oxidation peak of the Li_2_Mo_0.90_Co_0.10_O_3_ at 2.863 V is lower than that of Li_2_MoO_3_ and the reduction peak of the Li_2_Mo_0.90_Co_0.10_O_3_ at 2.136 V is above that of the pristine sample. Therefore, Li_2_Mo_0.90_Co_0.10_O_3_ possesses a smaller difference of the redox peak potential (∆E, 0.727 V) than Li_2_MoO_3_ (0.987 V). It is well known that difference of the redox peaks potential is highly correlated with electrode polarization. Hence, the conclusion can be drawn that Co-doping can reduce the polarization of Li_2_MoO_3_, which are coincident with the improvement in electrochemical performances. 

**Figure 10:**
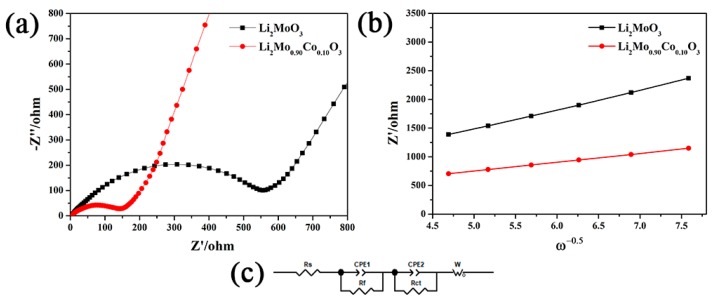
(**a**) EIS plots of Li_2_MoO_3_ and Li_2_Mo_0.90_Co_0.10_O_3_ after 20 cycles between 1.5 and 4.5 V at 5 mA·g^−1^, (**b**) *Z*′ vs. ω^−0.5^ at low frequency of EIS plots and (**c**) equivalent circuit model.

To further analyze the kinetic behaviors of the pristine and Co-doped samples, EIS measurements of Li_2_MoO_3_ and Li_2_Mo_0.90_Co_0.10_O_3_ were carried out and the results are presented in Figure 10. Both EIS plots display similar shapes (see Figure 10a), which are fitted through the equivalent circuit (see Figure 10c) and the fitting results are listed in Table 1. In the equivalent circuit, *R_f_* is related to Li^+^ diffusion in the SEI film, *R_ct_* is corresponding to the charge transfer resistance at electrolyte-electrode interface and *R_s_* is considered to be ohmic resistance. As can be seen from Table 1, the *R_s_* and *R_f_* of both samples change slightly. In contrast, the *R_ct_* of Li_2_MoO_3_ is significantly reduced duo to the Co-doping. The Li_2_Mo_0.90_Co_0.10_O_3_ presents the *R_ct_* of 105.70 Ω, which is far below the pristine Li_2_MoO_3_ (478.75 Ω). The *R_ct_* is major part to the total electrode impedance, and its reduction reveals that Co-doping is very beneficial to enhance the kinetic behaviors of Li_2_MoO_3_. In addition, The Li^+^ ion diffusion coefficients (*D_Li_*^+^) were estimated by the following formula:

(1) $$D_{Li^{+}}=\frac{R^{2}T^{2}}{2A^{2}F^{4}C_{Li^{+}}^{2}\sigma^{2}}$$

*R*, *T*, *A*, *F* and *C_Li_*^+^ are the gas constant, the absolute temperature, the area of the electrode surface, the Faraday’s constant and the molar concentration of Li ions, respectively [7]. The σ corresponding to the Warburg factor could be calculated by the *Z*′/ω^−0.5^ (see Figure 10b) and the following formula:

(2) $$Z^{\prime }=R_{f}+R_{ct}+{\sigma \omega }^{-0.5}$$

The improving trend in the values of *D_Li_*^+^ is very similar to the reducing trend in the values of *R_ct_*. The pristine and Co-doped Li_2_MoO_3_ deliver the Li^+^ ion diffusion coefficients of 3.89 × 10^−17^ and 1.94 × 10^−16^ cm^2^·s^−1^_,_ respectively. As we can see, the Li^+^ ion diffusion coefficients of Li_2_MoO_3_ have an obvious growth due to the Co-doping. These results clearly indicate that Co-doping significantly improves the kinetics behavior of Li^+^ and reduces the impedance of Li_2_MoO_3_, which is responsible for the better rate capability and cycling stability of Li_2_Mo_0.90_Co_0.10_O_3_.

**Figure 11:**
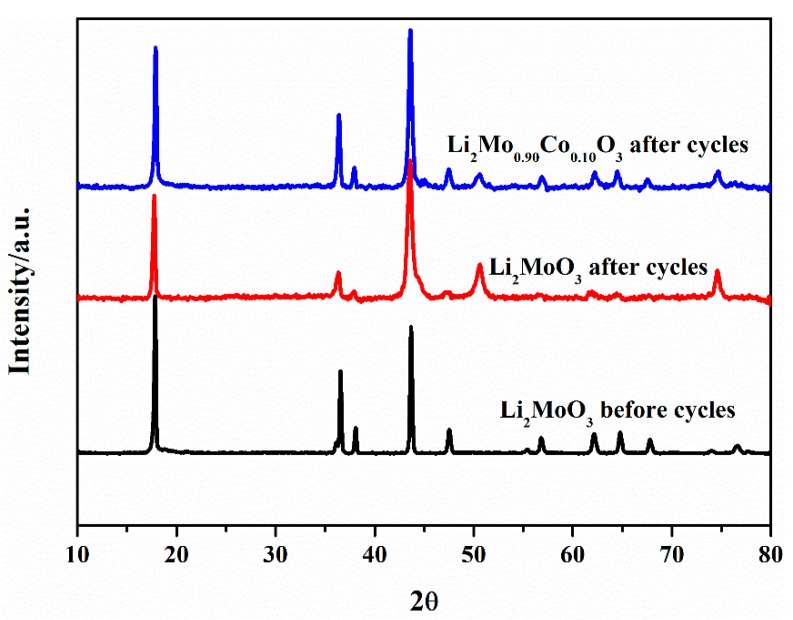
XRD patterns of Li_2_MoO_3_ and Li_2_Mo_0.90_Co_0.10_O_3_ after 20 cycles.

To investigate the structural transformation of the samples during charge-discharge process, XRD patterns of Li_2_MoO_3_ and Li_2_Mo_0.90_Co_0.10_O_3_ after 20 cycles are exhibited in Figure 11. There are notable differences in the structure of Li_2_MoO_3_ before and after cycling. For pristine Li_2_MoO_3_ material, the strongest diffraction peak transfers from (003) to (104) and ratio of (003)/(104) drops to 0.65 after cycling, which could be indexed into the Li-insufficient structure [17]. This Li-insufficient structure contributes to the partially reversible migration of the Mo ions and the partial recovery of the Mo_3_O_13_ clusters during charge-discharge processes, leading to irreversible capacity loss and poor cycling stability. In contrast, ratio of (003)/(104) of Li_2_Mo_0.90_Co_0.10_O_3_ after cycling can maintain at 0.83, which is much better than the pristine Li_2_MoO_3_ after cycling. It indicates that Co-doping can effectively enhance the structural stability during charge-discharge process.

## 4. Conclusions

Co-doped Li_2_MoO_3_ was successfully synthesized via a solid phase method. The influences of Co-doping on the structural and electrochemical characteristics of Li_2_MoO_3_ are analyzed. The results show that the addition of Co affects the morphology and decreases the particle size of cathode materials. Electrochemical measurements confirm that Co-doping can effectively improve the electrochemical performances of Li_2_MoO_3_ materials. The Li_2_Mo_0.90_Co_0.10_O_3_ presents an initial discharge capacity of 220 mAh·g^−1^ with the capacity retention of 63.6% after 50 cycles at 5 mA·g^−1^, which is much better than the pristine samples (181 mAh·g^−1^, 47.5%). Additionally, the rate capability of Li_2_MoO_3_ is also enhanced by Co-doping. It is found that the Li_2_Mo_0.90_Co_0.10_O_3_ delivers a much lower *R_ct_* and a higher Li^+^ ion diffusion coefficients than pristine Li_2_MoO_3_. What is more, the irreversible structural transformation is also suppressed by Co-doping. The enhanced electrochemical performances could be attributed to the improvement in structural stability and reduction in impedance due to the Co-doping. Our work reveals that doping modification will be a promising method for improving the electrochemical performances of Li_2_MoO_3_ material, and thus benefit for its application.

## Figures and Tables

**Table 1:** Fitting results of EIS plots.

Samples	*R_s_*/Ω	*R_f_*/Ω	*R_ct_*/Ω	*D_Li_*^+^ (cm^2^·s^−1^)
Li_2_MoO_3_	6.21	32.73	478.75	3.89 × 10^−17^
Li_2_Mo_0.90_Co_0.10_O_3_	5.89	25.79	105.70	1.94 × 10^−16^
